# Trochoidal Milling and Neural Networks Simulation of Magnesium Alloys

**DOI:** 10.3390/ma12132070

**Published:** 2019-06-27

**Authors:** Ireneusz Zagórski, Monika Kulisz, Mariusz Kłonica, Jakub Matuszak

**Affiliations:** 1 Department of Production Engineering, Mechanical Engineering Faculty, Lublin University of Technology, 20-618 Lublin, Poland; 2 Department of Enterprise Organisation, Management Faculty, Lublin University of Technology, 20-618 Lublin, Poland

**Keywords:** milling, the cutting force components, vibrations, magnesium alloys, artificial neural networks

## Abstract

This paper set out to investigate the effect of cutting speed v_c_ and trochoidal step s_tr_ modification on selected machinability parameters (the cutting force components and vibration). In addition, for a more detailed analysis, selected surface roughness parameters were investigated. The research was carried out for two grades of magnesium alloys—AZ91D and AZ31—and aimed to determine stable machining parameters and to investigate the dynamics of the milling process, i.e., the resulting change in the cutting force components and in vibration. The tests were performed for the specified range of cutting parameters: v_c_ = 400–1200 m/min and s_tr_ = 5–30%. The results demonstrate a significant effect of cutting data modification on the parameter under scrutiny—the increase in v_c_ resulted in the reduction of the cutting force components and the displacement and level of vibration recorded in tests. Selected cutting parameters were modelled by means of Statistica Artificial Neural Networks (Radial Basis Function and Multilayered Perceptron), which, furthermore, confirmed the suitability of neural networks as a tool for prediction of the cutting force and vibration in milling of magnesium alloys.

## 1. Introduction—State-Of-The-Art 

The aircraft and automotive industries demand innovative modern components and parts of uncompromising quality, which would be unattainable without employing innovative materials and high-efficiency machiningemploying advanced tools and machining centres. In order to fulfil the challenges of the modern market, manufacturers often face substantial production costs; therefore, engineers are relentless in their striving for the optimisation of production costs while maintaining high quality. In order to enhance the stability and effectiveness of subtractive machining, a thorough analysis of real cutting forces and computer simulation are indispensable. Artificial neural network simulation continues to confirm its applicability as an effective cost-optimisation solution, primarily by limiting the time and amount of testing required to specify and optimise machining parameters, which consequently leads to minimising errors in machining. 

In the search for optimisation in manufacturing, it is the selection of production materials that may eventually prove to play the leading role. The use of magnesium alloys significantly contributes to reducing the design weight and boosts savings in production and maintenance costs. On the other hand, the use of Mg alloys also has a beneficial effect on an inherent component of machining—effective removal of machining allowances—which is often carried out by means of milling. Forces occurring in machining are critical to the performance and quality of the process itself, which is why they are typically considered among the key “functional” machinability parameters. With respect to “physical” and additional absolute machinability parameters, the following may be distinguished; damping characteristics of materials, the amplitude and frequency of vibrations. In unfavourable conditions, different the cutting force components acting on the workpiece may cause uncontrollable deformation of the workpiece during machining, e.g., leading to the reduction of the undeformed chip thickness, which subsequently results in the increase in the cutting force. Then again, as a result of the increased shear energy per unit of volume, both the amount of subtracted workpiece material and the value of cutting forces increase [1]. Adhesion and build-up may similarly cause fluctuation in the components of the cutting force and cause insufficient surface finish quality or shape and dimensional accuracy. Compared to the machinability of other materials, magnesium alloys are characteristically quick and effective to cut, which enables executing the process at large depths of cut and high feed.

### 1.1. The Cutting Force Components 

The analysis of the components of cutting forces is virtually indispensable in subtractive machining for medical and aircraft purposes—the components implemented in these industries must meet particularly severe quality requirements. This is even more difficult given the frequently manufactured thin-walled workpieces of complex geometry [2,3,4]. Considerable cutting forces could potentially decrease the surface finish quality, as a consequence of excessive chatter in the milling machine-milling cutter–workpiece–fixture system [5]. Moreover, adhesion and build-up are similarly considered to be detrimental to the total cutting force. They are a source of major fluctuations of the cutting force components during cutting, and, as a result, deteriorate the condition of the surface finish of the final piece as well as its dimensional and shape accuracy [6]. 

Tool geometry is found to strongly affect the total cutting force and the amplitudes of its components. A study including Kordell geometry tools showed that the F_x_ and F_y_ components as well as their amplitudes rise in response to growth in feed per tooth f_z_. In addition, in the case of tools of classic geometry, the cutting forces and their amplitudes tended to respond more to the modification of feed per tooth f_z_ than to the cutting speed v_c_; the results from the study showed that the cutting force components peaked in machining with PCD tools and AZ91HP alloy. Finally, the cutting force components were shown to drop with the cutting speed was raised to v_c_ = 1200 m/min when employing traditional-geometry tools [7]. Research studies in the field of the cutting force components and their amplitude tend to focus on their response to particular technological milling parameters, such as v_c_, f_z_ and a_p_. Kuczmaszewski and Pieśko’s study investigated the milling process performed with carbide cutters of variable tooth geometry (γ = 5° and 30°). It showed that the cutting force components and their amplitudes—both important indicators of cutting stability—were shown to decrease when cutting with the tool γ = 30°. Furthermore, the cutting forces in milling exhibited great dependence on the cutting tool coating material (here: TiB2 and TiAlCN). The lowest values of the cutting forces (F_x_, F_y_) in milling Al6082 aluminium alloy were recorded when cutting with TiB2-coated tools. What is more, a typical transition point was reflected in the measurement data when v_c_ reached the high-speed cutting levels (v_cgr_ = 450 ÷ 600 m/min). In addition, the cutting force component amplitudes—relevant from the viewpoint of the cutting process dynamics—have been reported to attain the highest values for indexable tools, which are due proper consideration when selecting tools for particular applications [8]. 

In the study by Monies et al. [9], extremely deep pockets were cut in MRI301F (Mg-Nd-Y-Zr-Zn) workpiece, by means of two methods: plunge milling and BotTCF ramping at successive depth levels. The cutting force components were derived from test data. The maximum values of F_t_, F_r_ and F_a_ were as follows; in zone 1 (the start and end of each trajectoryone cutting tooth submerging in the workpiece): F_t_ = 1136 N, F_r_ = 450 N and F_a_ = 253 N—cutting force of 1248 N; in zone 2 (two teeth cutting): F_t_ = 572 N, F_r_ = 330 N and F_a_ = 468 N—cutting force F_Rmax_ = 827 N. Maladjusted cutting forces are the source of plastic strain in workpiece and generally increase the thickness of the undeformed chip, which, consequently, affects the shear angle and results in higher temperature in the area of cut. On the other hand, it was found that the lower the undeformed chip thickness, the higher the cutting forces [1].

Interesting insights into the behaviour of the cutting force components are found in works of Oczoś, who investigated the process of AZ91HP alloy milling with fluid by means of PCD cutters. It was shown that the cutting force components remain low and grow steadily with feed, which is of considerable significance to tool wear. Lower cutting forces curb heat, owing to reduced friction in the cutting zone. Furthermore, especially with respect to layers subtracted at a small radial depth of cut, the temperature in the area of cut is reportedly smaller [6]. PCD-cutter tools have been widely reported to produce better quality of surface finish, generate lower cutting force values and prevent tool overheating by decreasing friction at tool–workpiece interface [10]; consequently, the temperatures in the area of cut are significantly lower [11,12,13]. The cutting tool was the subject of analysis in the study by Zagórski et al. [14,15], which investigated the AZ91HP magnesium alloy machining with “serrated”, i.e., “wavy-edged”, cutting tools. In the study, the cutting forces and amplitudes were considerably higher compared with classic-geometry tools, whose amplitudes and components moreover exhibited higher sensitivity to modification of feed per tooth f_z_ rather than the cutting speed v_c_. While the primary objective of the study by Shi et al. [16] was to assess how tool wear affects cutting performance and to investiagte the wear mechanism of carbide inserts (in dry milling at speeds ranging from 1600 to 2000 m/min); it also provided data on the behaviour of the cutting forces. The analysed mechanisms were: abrasion, adhesion wearand diffusion. It was shown that prior to tool damage at speeds amounting to 1800 and 2000 m/min, extensive flaking appeared at the speed of 1600 m/min. The conclusion from the study was that increased tool wear causes the cutting forces to rise—resulting in the rise in cutting resistance. 

There is a well-evidenced tendency showing that the cutting force components largely depend on the cutting process conditions, as concluded by Sivam et al. [17], whose study involved the analysis of cutting forces and their response to selected input parameters (i.e., depth of cut, feed per tooth, spindle speed and tool diameter) recorded in ZE41 magnesium alloy dry face milling. The cutting forces were reported to increase gradually when the input parameters increased and, furthermore, they were to a greater extent affected by the change in spindle speed than in feed per tooth. Among the conclusions from the study, the relevant one from the point of view of behaviour of cutting forces was that there is a proportional relationship between the constant depth of cut and tool diameter and the total cutting force when operating at higher spindle speeds and feed per tooth respectively; moreover, it was evidenced that by monitoring cutting forces we may prolong tool life. 

In conclusion, there are numerous factors that exert a direct and indirect impact on the total cutting force, such as the type of tool and the material of the workpiece, tool geometry or perhaps, most essential, cutting parameters. Machining is more efficient when executed at higher speeds, feed rates and depths of cut [18,19,20]. Simultaneously, due to the fact that the relationships between the said factors show a nonlinear character, there is a growing interest in the application of mathematical analytical methods [2,18,21,22] or artificial intelligence systems [15,23,24,25] in modelling machining processes.

The main objective when modelling nonlinear milling processes is to predict the technological process, which could subsequently lead to developing a decision-making support system for an enterprise (e.g., optimising the milling process by determination of suitable machining parameters). The cutting forces are typically modelled with artificial neural networks (ANNs), with the application of e.g., Statistica software, which offers a selection of available network solutions, such as RBF (Radial Basis Function) and MLP (Multilayered Perceptron), which were, e.g., employed in the study by Zagórski et al. [15]. The authors investigated a simple milling process model including two variable parameters, cutting speed and feed per tooth, whereas others—axial depth of cut, radial depth of cut and helix angle—were fixed. The modelled machining process tested the effect of change in v_c_ and f_z_ at the same time eliminating the need for further real-life testing. Mathematical modelling was also included in Wu et al. [26], which showed a good fit of results from tests and simulations. The model for predicting the cutting forces was developed for trochoidal milling and enabled predicting the curvature effects of tool path on chip thickness and the impact of entry and exit angles on the process itself.

This section briefly introduced the problem of the cutting force components through relevant literature. Monitoring forces in cutting is critical to obtaining desired milling effects. Numerical simulation of nonlinear machining processes exponentially aids this analysis, particularly with respect to the investigation of optimal machining parameters at limited knowledge.

### 1.2. Vibrations (Displacement and Acceleration)

Vibration is typical for milling, which significantly affects the quality of finish and is decisive for maintaining specified parameters of the product. Due to its great relevance for the process, it is considered as one of the additional machinability parameters in milling. This relevance is, however, a negative one, i.e., it is highly undesirable during machining, particularly due to chatter occuring at the tool–workpiece interface (due to immediate surface disproportions). Vibrations heavily contribute to poor finish, dynamic tool wear (the consequence of high friction and temperature in cutting zone) [12,13] or decrease in machining accuracy and even damage of machine tool components. Surfaces that are predominantly subject to accelerated wear are those in permanent or temporary joints or actively involved in machining [27]. Moreover, vibrations in milling deteriorate machining efficiency and may even disrupt the entire production process, not to mention the fact that they may put the machine operators’ health at risk. Another reason why vibration is highly undesirable in production is the randomness of the end product characteristics (i.e., fulfilling target dimensions, shape and surface finish). Vibrations are even less acceptable in finishing [28] and milling thin-walled structures [29,30,31]. It is for these reasons that the stability of the process is of utmost importance. In an attempt to ensure it, engineers and researchers typically employ stability lobe diagrams (or the analysis of recurrence quantification), which effectively determines stable machining regions for workpieces of decreasing (nonuniform) wall thickness.

It seems then that the key question in milling isetermination of machining conditions imparting stability by preventing vibration [32,33], which is in fact frequently performed with the application of various specialist software. Milling parameters that are instrumental to machining stability are spindle speed n and axial depth of cut a_p_. Modern sophisticated innovative cutting tools, machine tools and materials highlight the need for controlling and modelling the mill cutting process. The unending race for efficiency and cost-effectiveness fuels the development of new methods that would resolve the problem of the cutting tool and workpiece vibration [21].

Although highly undesirable, vibrations are an inherent element of various technological operations, and are thus impossible to eliminate. Their intensity is predominantly the consequence of the machining conditions, tool wear, inconsistency of environmental conditions or workload. Vibration may be successfully curbed and maintained; however, it is essential to possess information regarding the work of particular machine elements and how they interact with each other [21,28].

A range of chatter prevention methods have been established: numerical simulation and modelling the process dynamics, the use of automation devices, pulsating frequency of spindle speed or work at high rates of feed per tooth. The study by Quintana et al. [34] distinguishes two major groups of vibration prevention methods, which embrace: determination of stable cutting parameters from lobe diagrams (group one) and methods enabling the change of system behaviour and adjustment of stability lobes (group two).

In the study by Weremczuk et al. [27] the active chatter control in open and closed loop consisted in the introduction of an external force. The numerical analysis executed in MATLAB–Simulink employed the fourth-order Runge–Kutta method with variable step of integration into the simulation of a nonlinear model of two degrees of freedom, which accounted for the phenomena accompanying the process, the susceptibility of the tool for nonlinearity and harmonic motion of the workpiece in the feed direction. Stable machining conditions and the value of the external excitation of the workpiece were derived from stability lobe diagrams. It was shown that the determined parameters denoted unstable machining. In the case of the closed-loop system, the control is executed by means of a proportional integrating PD controller, and it was the combined effect of external forces, suitably adjusted machining parameters and the controller in question that produced the desired effect of chatter reduction.

An alternative approach for stability analysis was employed in Wu et al. [31], which combined phase plane method, Poincare method and spectral analysis. The researchers explored the relationship between the maximum Lyapunov exponent, and the changes in milling depth and spindle speed, which allowed them to determine stable milling conditions in contouring. The maximum Lyapunov exponent of vibration signals, established as the stability criterion, was shown to increase with the rising depth of cut. Furthermore, the maximum Lyapunov index equal to 0.61 was selected as a threshold value of milling chatter nonlinear criterion. The study has, therefore, concluded that the maximum Lyapunov exponent of the workpiece vibration signal may be employed in the processing parameters optimisation.

The most relevant factors to the stability of milling are: machining strategy, machine tool, the cutting tool and its mounting system, chucks, the workpiece, cooling and lubrication and cutting data. Due to their specific machinability characteristics, resulting in their susceptibility to chatter during milling, thin-walled aluminium alloys, such as EN AW-7075 T6 [31,35], EN AW-2024-T351 [36] and EN AW-6061-T6 [30,37], typically undergo stability analyses. Rusinek et al. [35] investigated the cutting forces and acceleration to specify stable machining conditions for workpieces withdecreasing wall thickness. The analysis was performed by means of the recurrence plots and also involved recurrence quantification analysis (RQA), and showed that it is crucial for the determination of stability diagrams are variations in the modal cutting parameters for thin-walled features.

Vibration in the area of cut can also be modelled by means of finite element method (FEM) simulation. The scientific literature in the field presents different approaches, such as the one given by Yang et al. [30]. The proposed model of tool–workpiece system accounts for its dynamic behaviour as well as the effect of tool engagement and direction of feed. The numerical and experimental results proved that chatter could be accurately predicted for peripheral milling of thin-walled workpieces. In a different study, by Yusoff [37], another improving stability analysis parameter was considered, namely the root mean square (RMS) of vibration displacement, which was employed in the investigations of the positive effect of varying angle of inclination of the helix on the modulation of regenerative effect and thus on vibration damping. FEM was also applied in simulation works conducted in milling EN AW-2024-T351 alloy, and was shown to aid the determination of tool vibration effect on chip morphology, cutting force, and surface topology. In the referred study, by Rusinek and Zaleski [35], chip morphology was predicted under dynamic cutting conditions with the application of a hybrid dynamic cutting model (HDC). The data obtained from the numerical analysis showed that cutting speed and chip thickness increase vibration. Milling process limit stability is also determined by the use of methods based on constant training algorithms. The works reported in Friedrich et al. [38] introduced a novel method for the assessment of process stability, carried out by measuring acceleration signals. The paper introduced a new criterion providing information on the prediction assessment for a given input region—the multidimensional stability lobe diagram (MSDL). The research works were conducted with the application of a support vector machine (SVM) and ANNs. Whereas in the study by Olvera et al. [39], the stability charts were obtained from enhanced multistage homotopy perturbation (EMHP) method, which accounts for the impact of the helix angle and, what is essential, of the runout and cutting speed in machining of Al 7075 T6. As a result, thus obtained stability boundaries show a significantly improved accuracy.

Another work, by Munoa et al. [40], overviewed chatter damping techniques from the perspective of machinability and stiffness of workpiece material in the machine–tool–workpiece–fixture system. With the view to ensuring process stability, the work employed stability lobe diagrams. SLD-based (stability lobe diagrams) analyses, such as in the study by Hsiao and Huang [41], are applied with the purpose of investigating the relationships between the tool diameter chatter generation in milling. The study concluded that stability lobes increase withthe cutter diameter. It was, also shown that increasing the number of cutter teeth tends to move SLDs to the right (increased range of n).

Despite the numerous advantages, there are certain drawbacks of the SLD technique. The SLD data is derived from machine tools in their downtime. As a result, there are factors that cannot be accounted for in the analysis, such as the rigidity of the spindle system, the effect of excessive heat of machine tool parts in operation and other workpiece-related factors (e.g., mass-related damping properties of the workpiece, material defects or the variable diameter of cut) [40,42]. It is important that the obtained SLD results be cross-referenced with the cutting data from dynamic milling tests.

A different approach is employed by Campa et al. [43], which presents the process of calculating the stability diagrams that establish stable spindle speeds during the machining of thin floors. The study employs the milling model of the process carried out in the tool axis direction with bull-nose end mills. The model is combined with an analysis of modal parameters variation for machining thin floor workpieces, which consequently provides data for the calculation of the stability diagrams.

Typical chatter prevention and damping methods consist in either adjustment of technological cutting parameters, or restructuring the design of the machine tool. However, their common drawback results from the fact that both these classic methods are reactive rather than predictive, and respond to vibration observed in milling and not prevent it. From the perspective of implementation cost and time, modification of cutting data is more feasible, and the parameter that directly affects the entire process is spindle speed [44].

Milling nowadays is undergoing the speed revolution. High-performance cutting, which allows workpiece material to be efficiently removed, is slowly becoming a standard in machining. In HPC milling it is passive force component F_p_ that is key todiagnostics. The work by Burek et al. [45] investigated HPC milling of AlZn5.5MgCu (EN AW-7075) utilising the cutting force, acoustic emission and vibration signals. Excessive cost and complicated set-up requirements of modern dynamometers cause that alternative methods for the determination of the passive force component are developed, as exemplified by RMS acoustic emission or vibration measurement. The capacity of the former method consists in the high correlation between the signals of acoustic emission and the recorded values of the passive force component and, secondly, the acoustic method is sufficiently sensitive while being resistant to background noise. The major advantage of vibration amplitude measurement with accelerometer is that the obtained data describes the process dynamics while exhibiting good fit with the force data; on the other hand though, the method is sensitive to background noise interference.

ANN modelling of machining dynamics is yet to reach its full capabilities and scope of applications [38]. It may be regarded as a booster of efficiency, as it facilitates the selection of optimal technological parameters of milling [15,23]. Computer simulation is highly productive—it replaces long-term and costly experimental testing, thus limiting the involvement of cutters and machine tools. The models determine cutting parameters that guarantee minimum chatter, which is highly desirable in manufacturing performance and economy. Thanks to neural network simulations, initial process parameters are determined, along with their predicted outcome in the form of vibration [46].

### 1.3. Trochoidal Milling of Light Alloys

A range of CAM software applications enable executing trochoidal milling [47]: NX (Siemens, Munich, Germany), Catia (Dassault Systèmes, Vélizy-Villacoublay, France), hyperMILL (OPEN MIND Technologies AG, Wessling, Germany) and PowerMILL (Delcam, Birmingham, UK). The spiral tool path used in this method proves highly beneficial predominantly in difficult materials; nevertheless, with light alloys, trochoidal milling reduces cutting forces, vibrations and heat in the cutting zone [48,49].

Pleta et al. [50] investigated chip thickness and force modelling in trochoidal milling of 7075-T651 aluminium alloy. The novel approach to modelling chip thickness was employed in the low-to-medium range of cutting speeds (v_c_ ≈ 15–30 m/min). The immediate chip thickness was validated experimentally with the use of the semimechanistic cutting force model, exhibiting good fit of the simulated and experimental results. The cutting force coefficients for trochoidal milling were studied in another paper by the same author, [51], primarily to investigate the behaviour of machining parameters and coefficient values recorded for different tool paths. The tool feed and speed between trochoidal and slotting tool paths were found to produce large differences, which may be reduced given that the specified slotting parameters were derived from trochoidal milling chip geometry, with the closest agreement resulting from matching the maximum chip thicknesses. The 5-axis mill cutting was performed on 7075-T651 alloy, at n = 300–1450 rpm and v_f_ = 456–729 mm/min.

Otkur and Lazoglu [52] introduce an analytical model for defining the engagement along with the force model relative to engagement to determine machining forces. In addition, a numerical engagement model was developed. What is more, for the sake of trochoidal milling efficiency optimisation, the study investigates an alternative tool path strategy and double trochoidal milling. The resulting models were tested against the cutting results obtained from milling of Al 7039 alloy, executed at v_c_ = 22 m/min, v_f_ = 240 mm/min and a_p_ = 1 mm, only to show good correlation of the numerical and experimental results.

Rauch et al. [53] puts forward improvements for trochoidal tool path implementation in roughing. First, the maximum radial depth of cut according to tool path parameterisation was determined, which involved the use of two interpolation models. The second case was an improved tool path generation model for 5086 aluminium alloy pocket milling applications. As a result of the works carried out in the study, trochoidal tool paths were corrected according to objective process constraints. The following milling parameters (a 5-axis machine tool) were employed in the study; v_c_ = 2400 m/min, f_z_ = 0.35 mm/tooth, a_p_ = 6.66–13.50 mm and s_tr_ = 2–8 mm. The recorded maximal cutting force components obtained in the trochoidal milling tool path was equal to 3668 N, while 5670 N was recorded when following a zigzag tool path. The numerical analysis results were validated by the experimental tests. Although the models employed in the study in question appear to have overemphasised the radial depth of cut, according to the trochoidal model employed for programming the tool paths, they do, however, offer short calculation times, and are therefore suitable for quick tool path parameters selection and tool load evaluation.

Gao et al. [54] performed simulation analysis (with a 3D FEM model using Coupled Eulerian Lagrangian (CEL) to simulate milling) along with experimental validation on Al6061-T6 alloy. In the study, linear motion of the workpiece was adopted as a simplification of the trochoidal motion of the end mill. The machining was analysed for two axial depths of cut a_p_ = 0.3 and 0.5 mm, and three feed rate scenarios f_z_ = 0.04, 0.07, 0.10 mm/tooth. The cutting speed was constant for all cutting processes–n = 4500 rpm. The verification confirmed the usefulness of the model: the prediction error of the observed cutting forces can be maintained below 12%. The proposed model also shows high accuracy of predicted chip morphology. Unlike other 3D Lagrangian FEM models, the model put forward in this paper does not require a continuous remeshing algorithm to obtain high prediction accuracy. The described FEM model provides additional data, otherwise unextractable from classic cutting experiments, with respect to, e.g., contact forces and interfacial stresses.

Although magnesium alloy machining is a highly advantageous process, it involves several risks, such as the emergence of build-up, as a consequence of adhesion processes. Such conditions may contribute to the occurrence of variations in the components of the cutting force, which may consequently compromise the surface finish quality and the size and shape accuracy; secondly, they may lead to increased vibration and elevated temperature during the machining processes. Magnesium is widely known for its tendency for self-ignition, occurring when the temperature in the area of cut suddenly increases. Lastly, magnesium dust is a serious health hazard to machine tool operators and to the working of the machinery itself [6].

The cutting force components, vibration and computer simulations enhance the stability, security and efficiency of magnesium alloy machining. Mathematical models enable selecting optimal cutting parameters from the viewpoint of self-excited vibration, without engendering redundant machining errors. During milling, temporary surface irregularities may appear in response to chatter between the tool and the workpiece, and it is therefore absolutrly critical to determine stable machining conditions that will ensure a stable tool path and counteract vibration, and chatter. Simulation can be considered as a decision support tool for engineers with respect to specifying favourable technological parameters. ANNs have the capability to predetermine the particular machining parameters and predict vibration. The validation of numerical results provides positive evidence for further use of artificial neural networks for designing nonlinear processes. Finally, it appears that the scientific literature in the field of trochoidal milling is relatively scant, and therefore there emerges a necessity to investigate how to improve effectiveness, efficiency and stability of magnesium alloy milling.

## 2. Materials and Methods

The presented study was performed on two types of workpiece materials—AZ91D (MgAl_9_Zn_1_) and AZ31 (MgAl_3_Zn_1_) magnesium alloys—exhibiting high mechanical properties and very good corrosion resistance, which in turn earmark them for a number of different industrial applications. Die-cast magnesium alloys (e.g., AZ91D and Mg–Li) are found in a range of parts and elements manufactured for the aerospace and automotive industries, such as gear housing (e.g., of a steering gear), clutches, gears and components of combustion engines. Another type of magnesium alloys suitable for plastic forming applications (e.g., AZ31, AZ80 and WE43) can be used in aircraft elements, such as the cockpit control panel or transmission. The chemical compositions of the alloys are presented in Table 1.

All milling operations were performed on AVIA VMC 800 HS (Warszawa, Poland) vertical machining centre with Heidenhain iTNC 530 control (power 25 kW, n_max_ up to 24,000 rev./min and v_f max_ up to 40 m/min). The cutter tool was mounted in HSK 63A tool holder, capable of performing the cut at speeds from the high-speed machining range. With respect to cutting data, the range of changeable milling parameters was cutting speed v_c_ = 400–1200 m/min and trochoidal step s_tr_ = 5–30% of the cutter diameter and the fixed parameters of the process were feed per tooth f_z_ = 0.15 mm/tooth and axial depth of cut a_p_ = 6 mm.

Trochoidal milling operations were carried out with the straight shank solid carbide (VHM) end mill Fenes (Siedlce, Poland) with TiAlN coating, recommended for aluminium and magnesium milling applications. It is a 2-flute tool z = 2, diameter d = 16 mm of overall dimensions 16 mm × 25 mm × 100 mm and helix angle λ_s_ = 30°.

The cutting tool was mounted in SECO (Fagersta, Sweden) HSK63A SFD 16 × 120 thermal shrink-fit tool holder. Prior to mounting in the spindle, the tool holder system was balanced in CIMAT (Bydgoszcz, Poland) CMT-15V2N balancing machine, balancing class G 2.5 at 25 000 rev./min, according to ISO1940:2003, which permits a slight residual unbalance tolerance of 1 g mm/kg. In the study, the analysed milling cutter’s residual unbalance was 0.77 g mm.

**Figure 1:**
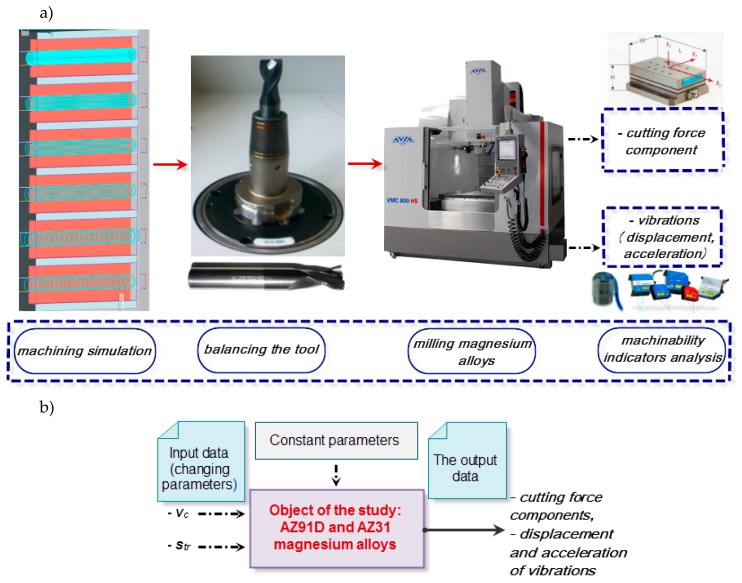
Schematic diagram (**a**) and test set-up (**b**) with the object of the study

Figure 1 shows the schematic diagram of the tested system (Figure 1a) and the test set-up (Figure 1b) with the object of the study. The model and the machining sequence were designed by means of NX 10.0–SIEMENS (Munich, Germany) software. The measurement apparatus applied in the study was, for cutting force measurement, piezoelectric dynamometer 9257B by Kistler (Winterthur, Switzerland); for vibration acceleration measurement, PCB 352B10 accelerometer by Piezotronics (New York, NY, USA); for vibration displacement, optoNCDT LD1605-2 laser sensor by Micro-Epsilon Masstechnik (Ortenburg, Germany); and the 3D surface roughness profiles were obtained from the T8000RC120-400 profilographometer provided by Hommel–Etamic Jenooptik (Jena, Germany).

In the reported tests, vibration displacement and acceleration measurements were performed in the Y-axis (along the machine tool axis). The cutting forces were measured with 9257B Kistler piezoelectric dynamometer and equipped with a charge amplifier 5017B. The techincal data for the Kistler dynamometer is presented in Table 2.

The basic specifications of the PCB 352B10 sensor are: sensitivity 1.02 mV/(m/s²), measurement range ±4905 m/s² pk, frequency range 2 to 10,000 Hz, resonant frequency ≥65 kHz and broadband resolution 0.03 m/s² rms. The specifications of the laser optical sensor optoNCDT LD1605-2 were measuring range 2 mm, resolution from 0.5 μm and spot diameter 0.3 mm. 3D surface roughness measurements were performed at the following specifications; Lt = 4.8 mm, Lc = 0.8 mm and v_t_ = 0.5 mm/s. The scanned sample surface area was 4.8 × 4.8 mm and it was measured in approximately 200 scanning steps.

Modal analysis is a process typically employed in various research works with the purpose of investigating basic dynamic parameters (i.e., parameters determined in milling, e.g., the cutting force components and vibration displacement/acceleration) of given systems as well as to generate stability lobe diagrams. The SLD analysis was performed with the use of CutPro software (9.0, Manufacturing Automation Laboratories Inc., Vancouver, BC, Canada), a 352B10 PCB accelerometer and a 070A02 PCB scope input adaptor.

**Figure 2:**
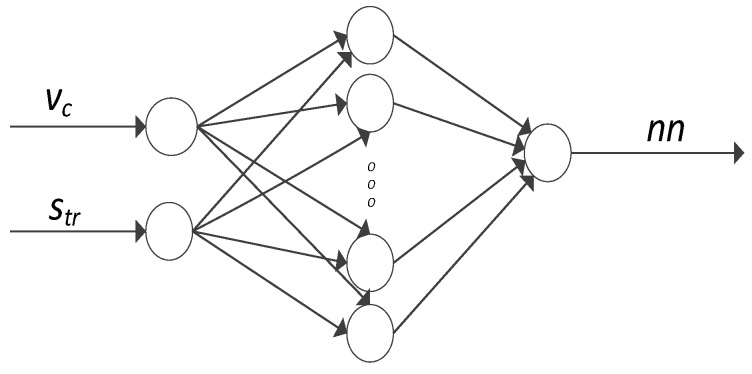
Schematics of the artificial neural network with the analysed process parameters

In addition, numerical simulation of process parameters was employed to predict the nonlinear technological process. It is rather difficult to define nonlinear processes by means of mathematical equations. Given that the milling process is a control object, then the simulated parameters, the cutting force components F_x_ and F_y_ and the vibration displacement (x) constitute its output parameters. The variable input parameters of milling were cutting speed v_c_ and trochoidal step s_tr_, while the remaining parameters were fixed. Figure 2 presents the schematic model of the process, where v_c_ and s_tr_ denote variable input parameters and nn gives the currently simulated corresponding parameter. Since there were three machining parameters and two workpiece materials analysed in the study—AZ31 and AZ91D—the total number of six different neural networks were obtained.

The network used in this study employed the black box model, as this solution offers good performance on the training set. Furthermore, the selected model is utilised whenever determination of mathematical equations describing the analysed process is complicated, as in the case with the process investigated in this study. It was thus resolved that the black box approach would most accurately reflect the complex character of milling. Since machining is repeatable, then the force input and cutting conditions in selected points of the tool path may be similarly considered repeatable. However, for the sake of accuracy of the model, it must be assumed that cutting is performed without tool wear and at constant cutting parameters in all consecutive machining cycles.

The cutting force components (F_x_ and F_y_) and vibration displacement in AZ31 and AZ91D alloy milling by means of artificial neural networks were modelled with the application of Statistica Neural Networks software. The network models employed in the study were RBF (Radial Basis Function) and MLP (Multilayered Perceptron). For both alloys, all machining parameters, the component F_x_ of the cutting force, the component F_y_ of the cutting force and the displacement of vibrations x were modelled separately. The established parameters were the average of the maximum values from the 10 ranges separated from the stable machining area and constituted the output value for individual models. The MLP network was trained with the application of the BFGS (Broyden–Fletcher–Goldfarb–Shanno) method, while the RBF network was trained with the RBFT. The former modelling method employed the linear, exponential, logistic, tanh and sinusoidal activation functions. The activation function of the RBF for hidden neurons is the Gaussian distribution and for the output neurons—a linear function. Both MLP and RBF networks contained one hidden layer, which was dictated by simplicity factors. In the input layer, there are two neurons (cutting speed v_c_ and trochoidal step s_tr_) and, in the output layer, the neuron represents a currently simulated parameter, i.e., F_x_ or F_y_ component of the cutting force or vibration displacement x). The number of training epochs was in the range of 150 to 250 epochs and the hidden neurons (2–10) were selected experimentally.

The key indicators of the network fitting were training quality, validation quality and training error determined with the least squares method. The learning group used 80% of measurement results and the remaining 20% of results served the purpose of validation. As postulated in the research by Zagórski et al. [46], given that the amount of available data is insufficient, the test group may be omitted, which is what occurred in the reported study.

## 3. Results and Discussion

**Figure 3:**
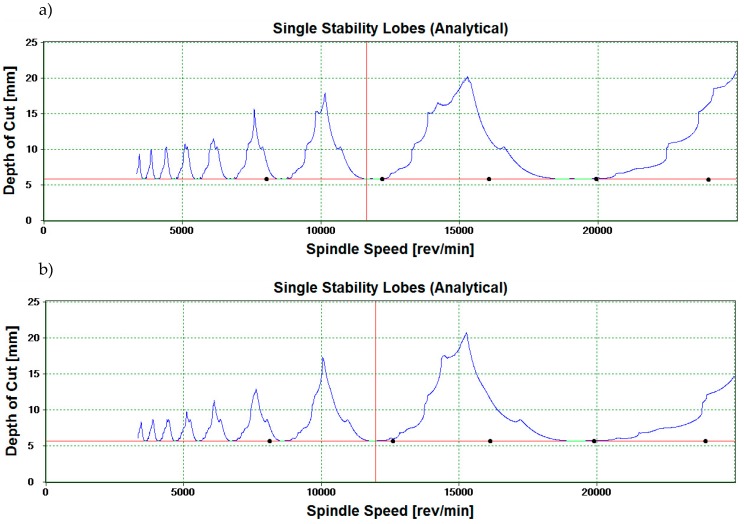
Stability lobes diagrams for (**a**) AZ31 and (**b**) AZ91D magnesium alloys

SLD analysis is a tool for the determination of stable machining areas. Operating within thus established parameters, i.e., in this case rotational speed n and axial depth of cut a_p_, should ensure that the system remains stable throughout milling. However, SLD analysis is carried out in static tests and fails to account for the dynamics of the machining system (resulting from the complexity of the machine tool and of the milling process itself). Figure 3 shows SLD diagrams for the tested cutting tool parameters and magnesium alloys.

The markings in the chart define the given machining regions as stable—in the upper part of the chart above the stability lobes (often marked as “+”), unstable—below the stability lobes (often marked as “–”)—and “•” the settings selected as milling parameters.

### 3.1. The Cutting Force Components

The components of the cutting forces F_x_, F_y_ and the displacement and acceleration of vibrations were recorded throughout the milling of magnesium alloys AZ91D and AZ31. The test results were presented in the form of box plot charts and bar charts showing the maximum values of the analysed machinability parameters. These charts provide the information regarding the maximum range of values of vibrations and of the total cutting force components, as well as the range, i.e., the difference between the minimum and the maximum values of the parameters in question. For certain machining settings, the charts also show outliers and/or extremes, which may be associated with the increase in the cutting force components and vibrations caused by temporary stability loss under given machining parameters. A tighter range of outliers/extreme values in box plots and lower values of standard deviation in bar charts may be indicative of improved stability of the process.

**Figure 4:**
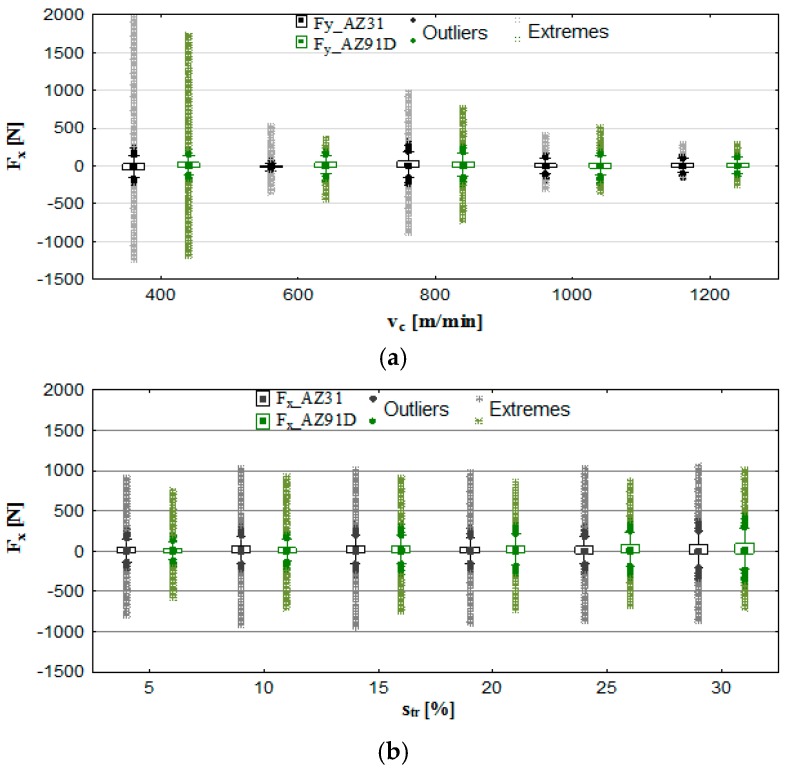
Effect of cutting speed and trochoidal step on cutting force component F_x_ in milling of magnesium alloys: (**a**) v_c_ (f_z_ = 0.15 mm/tooth, a_p_ = 6 mm, s_tr_ = 15%) and (**b**) s_tr_ (v_c_ = 800 m/min, f_z_ = 0.15 mm/tooth, a_p_ = 6 mm).

**Figure 5:**
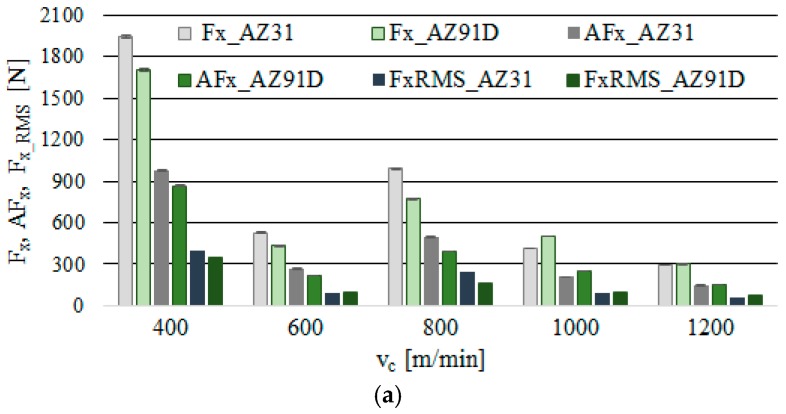

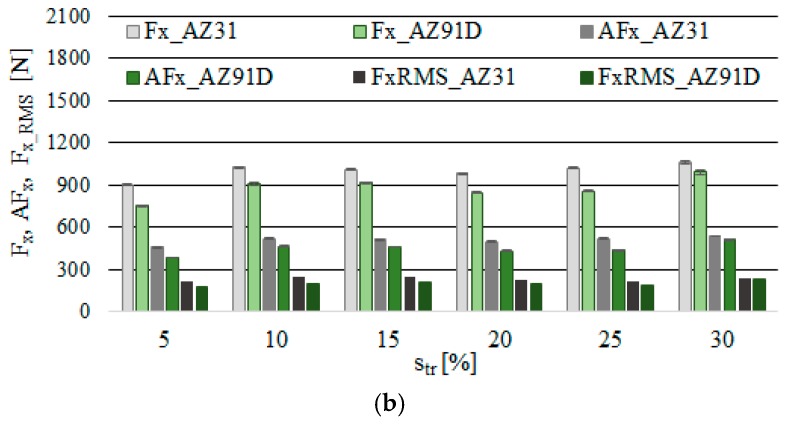
Maximum value of cutting force component F_x_, amplitude and RMS in the milling of magnesium alloys: (**a**) effect of cutting speed v_c_ (f_z_ = 0.15 mm/tooth, a_p_ = 6 mm, s_tr_ = 15%) and (**b**) effect of trochoidal step s_tr_ (v_c_ = 800 m/min, f_z_ = 0.15 mm/tooth, a_p_ = 6 mm).

**Figure 6:**
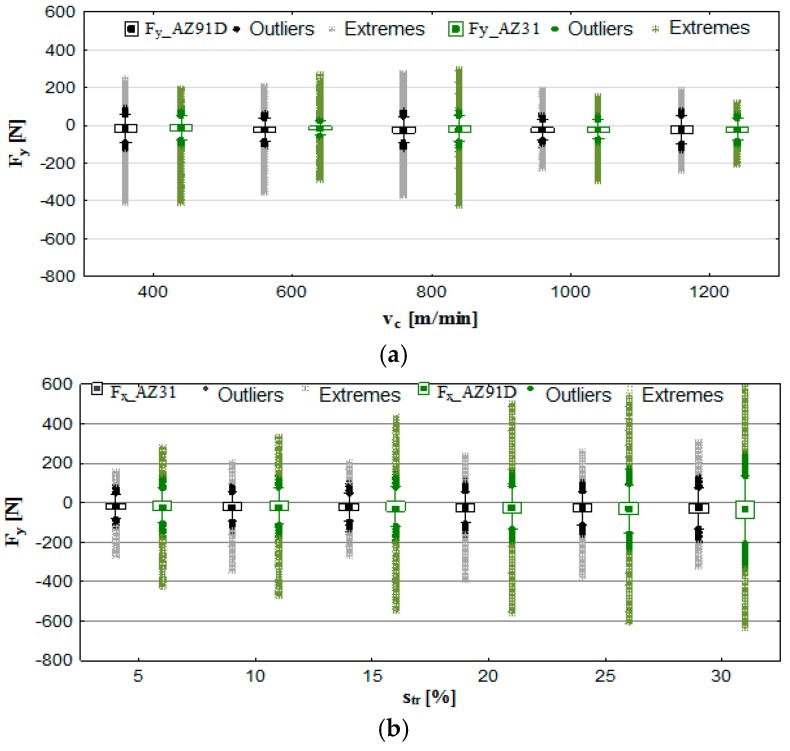
Effect of cutting speed and trochoidal step on cutting force component F_y_ in milling of magnesium alloys: (**a**) v_c_ (f_z_ = 0.15 mm/tooth, a_p_ = 6 mm, s_tr_ = 15%) and (**b**) s_tr_ (v_c_ = 800 m/min, f_z_ = 0.15 mm/tooth, a_p_ = 6 mm).

**Figure 7:**
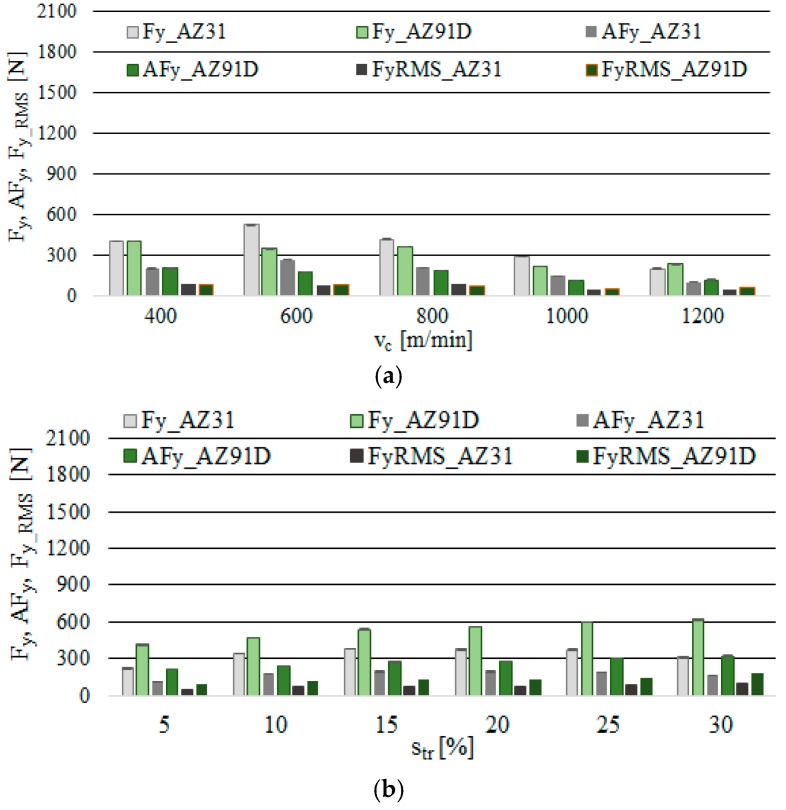
Maximum value of cutting force component F_y_, amplitude and root mean square (RMS) in the milling of magnesium alloys: (**a**) effect of cutting speed v_c_ (f_z_ = 0.15 mm/tooth, a_p_ = 6 mm, s_tr_ = 15%) and (**b**) effect of trochoidal step s_tr_ (v_c_ = 800 m/min, f_z_ = 0.15 mm/tooth, a_p_ = 6 mm).

Figures 4 and 6 show box plot charts, whereas Figures 5 and 7 show the maximum values of F_x_, F_y_, the amplitude of these components—AF_x_ and AF_y_—and the normalised force coefficient RMS values—F_x_RMS_ and F_y_RMS_. In addition, the standard deviation for individual components of the cutting force and its amplitude are presented.

It may be seen from Figure 4a that the most advantageous speeds from the perspective of the cutting force components are: 600 m/min, 1000 m/min and 1200 m/min, as it is under these conditions that the most beneficial conditions occur, i.e., minimum F_x_ and the tightest ranges of outliers and extremes. Extreme values tend to occur at v_c_ 400 m/min and 800 m/min. By analysing Figure 4b, it can be seen that trochoidal step change does not significantly affect the increase in the outliers and extreme ranges for the F_x_ component (over the entire s_tr_ change interval). Nevertheless, the change in the s_tr_ typically leads to the higher value of the F_x_ component (in the majority of cases) than in the change in v_c_. In conclusion, it is advantageous to carry out trochoidal milling within the scope of parameters pertaining to the HSM machining range. Similar to the use of straight tool paths [8], the components of the cutting forces drop with the increase in the cutting speed v_c_.

Similar tendencies as in Figure 4 are clearly visible in Figure 5, where the component F_x_, its amplitude AF_x_ and the normalised force coefficient RMS value F_x_RMS_ generally show a tendency to decrease practically over the entire tested cutting speed range (except for v_c_ = 800 m/min). Maximum F_x_ was recorded for the AZ31 alloy amounted to F_x_ = 1940 N and the minimum F_x_ = 291 N, in the AZ91D alloy: maximum F_x_ = 1707 N and minimum F_x_ = 301 N. In s_tr_ analysis, its values fluctuated in the range of 901 to 1020 N (for AZ31) and approximately 753 to 991 N (for AZ91D). Lower values of the F_x_ component were generally observed in AZ91D alloys.

On the basis of Figure 5, it can be observed that the normalised force coefficient RMS values exhibit a close correlation with the amplitudes: in the majority of cases, they constitute up to approximately 50% of this value. Upon a close investigation of the problem and having obtained the results from measurements, it may be concluded that the highest tool damage and workpiece deformation occur at the cutting speed v_c_ = 400 m/min and v_c_ = 800 m/min, whereas the smallest at v_c_ = 1200 m/min. It is, therefore, this machining region that should be indicated as “safe” considering the execution of milling processes, particularly in the case of thin-walled elements and parts (less machining deformations).

To an extent, Figure 6a appears to confirm the previous findings: more favourable machining parameters (as exemplified by fewer outliers and extremes) occur in the range of v_c_ = 1000 to 1200 m/min. However, in the case of trochoidal step (Figure 6b), the findings differ. Here the F_y_ component develops the minimum values for *p* = 5% of the cutter diameter. This is a consequence of the increase in the s_tr_ parameter and the simultaneous increase in the diameter of the area of cut. In this case, therefore, it is preferable to use high values of v_c_ speed and small trochoidal steps: s_tr_ = 5–15% for AZ31 alloy and *p* = 5–10% for AZ91D alloy.

The analysis of the maximum values, the amplitude and the normalised force coefficient RMS value of F_y_ (Figure 7) show that the aforementioned exhibit a similar response to the change in v_c_ as in the case of the F_x_. Here, the most favourable processing conditions were established as v_c_ = 1200 m/min, f_z_ = 0.15 mm/tooth, a_p_ = 6 mm and s_tr_ = 15%, for which the values of the analysed indicator were AZ31 alloy—F_y_ = 202 N, AF_y_ = 104 N and F_y_RMS_ = 50N, while in the AZ91D alloy—F_y_ = 236 N, AF_y_ = 120 N and F_y_RMS_ = 61N. Lower values of the F_y_ component of the total cutting force were typically observed: under changing v_c_ for AZ91D alloy, and in the case of the change in s_tr_ for AZ31 alloy.

### 3.2. Vibrations (Displacement)

With respect to vibration displacement, the analysed output data were: displacement of vibrations x, their amplitude Ax and the normalised force coefficient RMS value of vibration displacement x__RMS_. The analytical procedure for vibration displacement resembled that of the cutting force, i.e., the first stage consisted in computing the box plot charts, which subsequently provided the data for the bar graphs and next for determining the standard deviation. These relationships (x, Ax and x__RMS_) are presented in the function of the technological cutting parameter—cutting speed v_c_ and the additional parameter is trochoidal step s_tr_.

**Figure 8:**
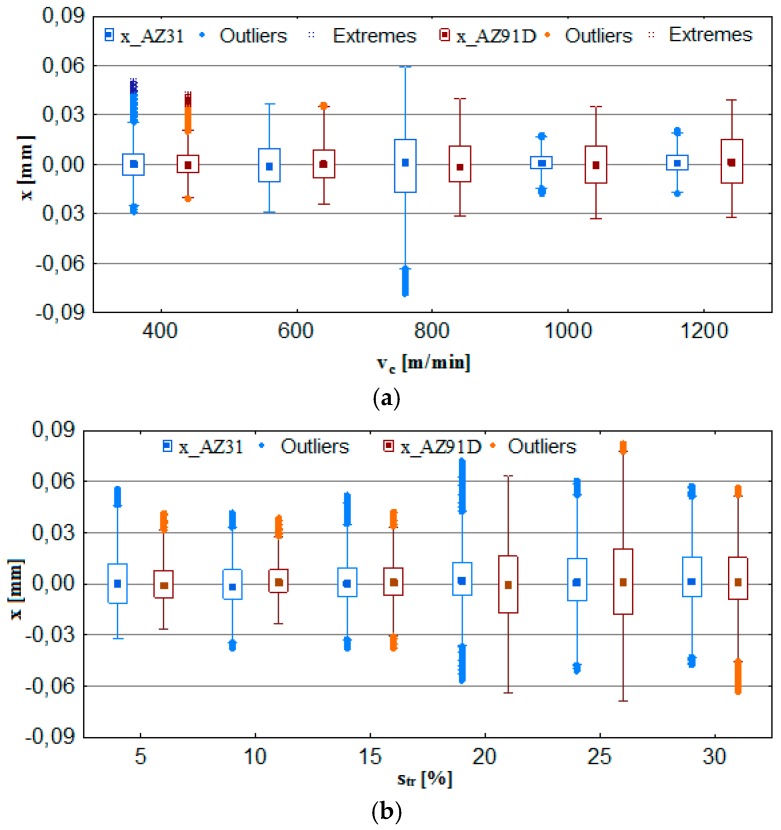
Effect of cutting speed and trochoidal step on vibrations displacement in milling of magnesium alloys: (**a**) v_c_ (f_z_ = 0.15 mm/tooth, a_p_ = 6 mm, s_tr_ = 15%) and (**b**) s_tr_ (v_c_ = 800 m/min, f_z_ = 0.15 mm/tooth, a_p_ = 6 mm).

Figure 8a indicates that the most favorable conditions regarding vibration displacement occur when milling is performed at v_c_ = 1000–1200 m/min. Considering AZ91D alloy, one may see no outliers or extremes, which clearly suggests very good machining stability throughout the entire range of cutting speeds v_c_. In the case of AZ31 alloy, although outliers/extremes are admittedly present in graphs, the box plot range is the smallest for the entire analysed range of v_c_. Figure 8b shows that trochoidal step change leads to an increase in the range of recorded values and outliers/extremes for vibration displacement. What follows from these observations is that it is more advantageous to repeatedly increase the cutting speed rather than the trochoidal step, due to the fact that higher values of v_c_ translate to a more favorable range of x. The most unfavourable range of displacement vibrations x occurs at s_tr_ = 20–30%.

**Figure 9:**
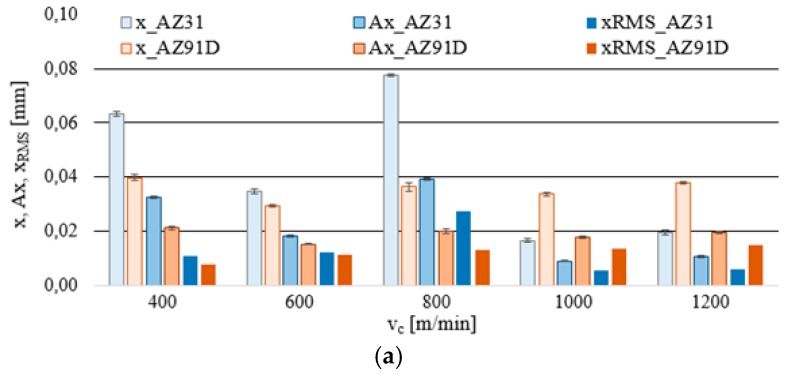

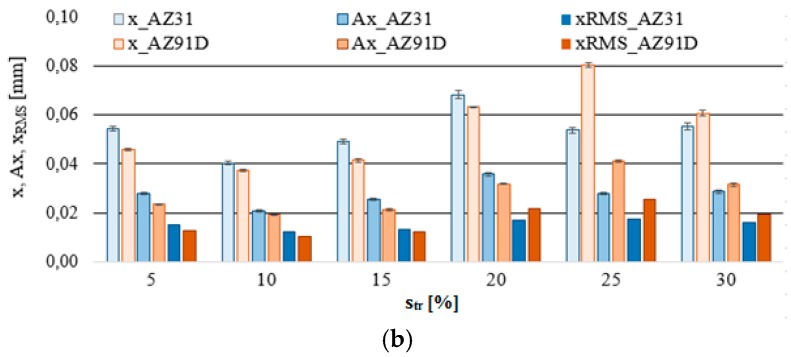
Maximum value of vibrations displacement, amplitude and RMS in the milling of magnesium alloys: (**a**) effect of cutting speed v_c_ (f_z_ = 0.15 mm/tooth, a_p_ = 6 mm and s_tr_ = 15%) and (**b**) effect of trochoidal step s_tr_ (v_c_ = 800 m/min, f_z_ = 0.15 mm/tooth and a_p_ = 6 mm).

As shown in Figure 9a, the most favorable cutting conditions are obtained for the speeds v_c_ = 600 m/min and v_c_ = 1000–1200 m/min. Comparable values of x, Ax and x__RMS_ (Figure 9b) were recorded, even for s_tr_ = 15%, however, at a lower cutting speed—v_c_ = 800 m/min. Therefore, the data suggest that in order to achieve an increase machining efficiency while not contributing to the deterioration of the machining conditions defined by the vibration displacement, it is more convenient to increase the cutting speed v_c_ rather than trochoidal step s_tr_.

Generally, it should be emphasised that vibration displacement values that can be regarded as “favourable” milling conditions do not exceed x = 0.04 mm. The normalised force coefficient RMS value of vibration displacement x__RMS_ is in the range of x__RMS_ = 0.005-0.012 mm.

### 3.3. Vibrations (Acceleration)

The analysis of the output vibration acceleration data was performed according to the procedure applied in the vibration displacement analysis. The selected output parameters were: vibration acceleration a, vibration acceleration amplitude Aa and normalised force coefficient RMS value of vibration acceleration a__RMS_. The results obtained from tests provided data for the computation of box plot charts and bar graphs with the standard deviation. Similarly to the formerly analysed parameters, the functional relations (a, Aa and a__RMS_) are presented in the function of the technological cutting parameter—cutting speed v_c_ and the additional parameter is trochoidal step s_tr_.

**Figure 10:**
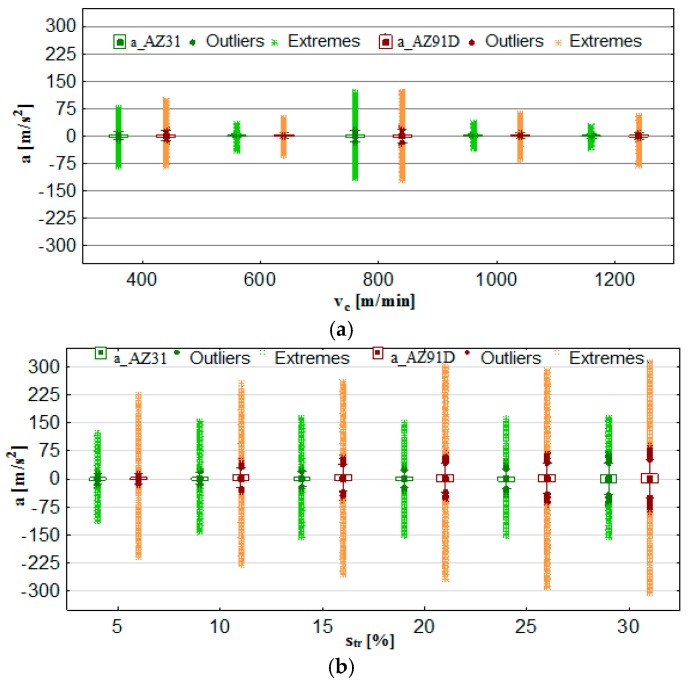
Effect of cutting speed and trochoidal step on vibrations acceleration in milling of magnesium alloys: (**a**) v_c_ (f_z_ = 0.15 mm/tooth, a_p_ = 6 mm, s_tr_ = 15%) and (**b**) s_tr_ (v_c_ = 800 m/min, f_z_ = 0.15 mm/tooth, a_p_ = 6 mm).

From Figure 10a, it may be seen that the most favourable cutting parameters with respect to vibration acceleration (and similarly to displacement analysis) are v_c_ = 600 m/min and v_c_ = 1000–1200 m/min. Under these conditions, the recorded vibration acceleration value does not exceed the range of ± 75 m/s^2^. A considerably higher range of values of vibration acceleration is observed in the trochoidal step change (Figure 10b). In general, outliers/extreme occur for all presented cutting conditions; however, the intensity of their occurrence is notably lower under the conditions of changing cutting speed v_c_.

**Figure 11:**
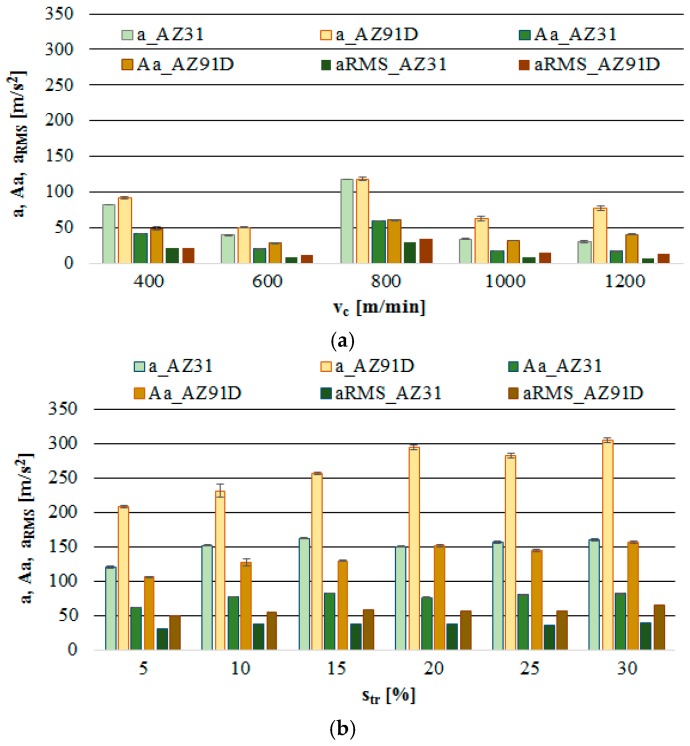
Maximum value of vibrations acceleration, amplitude and RMS in the milling of magnesium alloys: (**a**) effect of cutting speed v_c_ (f_z_ = 0.15 mm/tooth, a_p_ = 6 mm and s_tr_ = 15%) and (**b**) effect of trochoidal step s_tr_ (v_c_ = 800 m/min, f_z_ = 0.15 mm/tooth and a_p_ = 6 mm).

Figure 11a further confirms the previous observations regarding the most favourable cutting parameters as occurring at the speed range of v_c_ = 600 m/min and v_c_ = 1000–1200 m/min. At the given levels of v_c_, there are practically no instances in which the level of 50 m/s^2^ would be exceeded. The trochoidal step change (Figure 11b) results in the increase in a, Aa and a__RMS_. Therefore, it is in another instance that it becomes confirmed that machining efficiency is boosted by increasing the cutting speed v_c_. In this case, higher values of vibration acceleration were observed for the foundry alloy AZ91D.

### 3.4. 3D Surface Roughness

With the purpose of providing a more detailed analysis of milling effects and relationships between forces occurring in the process, selected 3D models of the machined workpiece surface roughness profiles are presented in Table 3, along with more important 3D roughness surface parameters. What is instantly visible is the characteristic lay of machining marks carved in the workpiece during each pass along the trochoidal tool path.

From the perspective of improving machining conditions with respect to surface roughness, the presented visual representation of the machined surface appears to indicate that higher cutting speed provides more favourable cutting conditions than higher trochoidal step value. In the presented example (cf. Table 3), the 3D roughness parameters are approximately 50% lower when machining at v_c_ = 1200 m/min, than for s_tr_ = 30%. This confirms the validity of thesis postulating that the increase in the cutting speed increases the efficiency and effectiveness of milling magnesium alloys. These values are typical for the surface obtained in the finishing milling proces (for s_tr_ = 30%) and comparable with rough grinding (for v_c_ = 1200 m/min).

### 3.5. Numerical Modelling of the Cutting Force Components and Vibrations with Artificial Neural Networks

The experimental data on the components of cutting forces as well as displacement of vibrations during milling of AZ91D and AZ31 alloys were utilised as input data for simulation of these quantities by means of suitable artificial neural networks. Simulations were carried out with the Statistica Neural Networks software and involved MLP and RBF networks.

There are several key network quality indicators assessing whether a suitable network type was selected: the quality of learning, the quality of validation as well as the learning error and validation error derived by means of the least-squares method. In the study, 200 networks were experimentally determined for each simulation; subsequently, the quality of networks was evaluated with the indicators above in order to select the most suitable alternative—MLP or RBF. The created network parameters for vibration displacement and the cutting force components F_x_ and F_y_ in milling of AZ31 alloy are given in Table 4. The analysis of the tested neural network models indicates that it is the RBF network that produces better results of simulation for all modelled parameters for AZ31 alloy: for vibrations displacement x the RBF neural network structure is 2-7-1 with seven hidden neurons, considering the F_y_ component the RBF network structure was 2-6-1 and, for the F_x_ component of the cutting forces, the network included five hidden neurons (RBF 2-5-1).

The MLP and RBG network parameters for vibration displacement are F_x_ and F_y_ and the cutting force components modelled in AZ91D alloy milling are presented in Table 5. The comparative analysis of the neural network models leads to the conclusion that for F_x_ component of the cutting force it was the RBF 2-5-1 network with five neurons that provided better results. However, with regard to other simulated parameters, better fit with the experimental data was obtained for MLP networks: the network with three neurons (MLP 2-3-1) for the F_y_ component of the cutting force, and with six (MLP 2-6-1) for vibrations displacement x.

**Figure 12:**
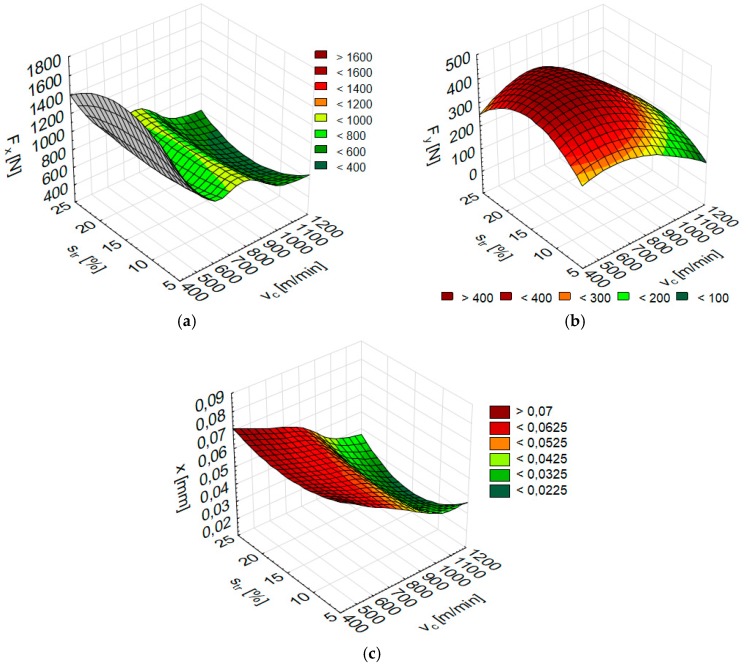
Numerical results of (**a**) Fx, (**b**) F_y_ and (**c**) x depending on the cutting speed v_c_ and trochoidal step s_tr_ for AZ31 alloy (F_x_: RBF 2-5-1, F_y_: RBF 2-6-1, x: RBF 2-7-1).

**Figure 13:**
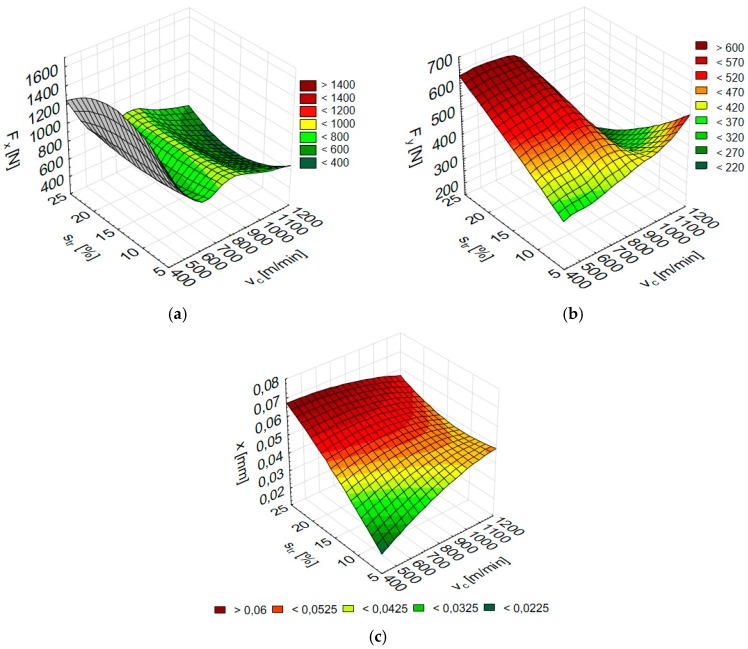
Numerical results of (**a**) Fx, (**b**) Fy and (**c**) x depending on the cutting speed v_c_ and trochoidal step s_tr_ for AZ91D alloy (F_x_: RBF 2-5-1, F_y_: MLP 2-3-1, x: MLP 2-6-1).

The numerical results of the total the cutting force components F_x_ and F_y_ and vibration displacement x according to the cutting speed v_c_ and trochoidal step s_tr_ for AZ31 substrate are shown in Figure 12, while for AZ91D alloy in Figure 13. Once v_c_ and s_tr_ are fed into Statistica, the corresponding parameters (F_x_, F_y_ and x) are obtained.

**Figure 14:**
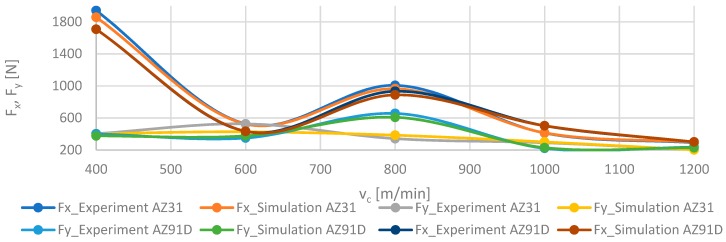
Comparison of experimental and numerical results depending on the cutting speed v_c_ for trochoidal step s_tr_ = 15% for F_x_ and F_y_ components of the cutting force in milling of AZ31 and AZ91D alloys.

**Figure 15:**
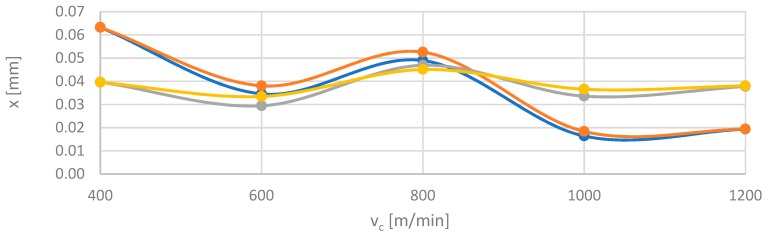
Comparison of experimental and numerical results depending on the cutting speed v_c_ for trochoidal step s_tr_ = 15% for vibrations displacement x in milling of AZ31 and AZ91D alloys.

The accuracy of the modelled networks is shown in Figure 14, which provides a comparison of F_x_ and F_y_ components of the cutting force for AZ31 and AZ91D alloy material with numerical values depending on the cutting speed v_c_ at fixed trochoidal step s_tr_ = 15%. Figure 15 presents a comparison of modelled and experimental data for vibration displacement x for the same parameters of both alloys. Analysing simulation results with actual values of simulated parameters, the relative error did not exceed 15%.

The numerical results obtained from simulations exhibit an acceptable level of error, below 15%. On the one hand, this is a result, and on the other, the indication of artificial neural networks suitability for the use as a tool for simulating, e.g., parameters of abrasive water jet machining. Such data becomes instrumental for the elaboration of working numerical models. The capabilities of artificial neural networks earmark them as a perfect tool for numerical modelling of machining processes.

## 4. Conclusions

The analysis of the experimental and mathematical data produced in the presented study leads to the formulation of the following conclusions.

1. Modification of either of the tested machining parameters, v_c_ or s_tr_ affects the values of the cutting force components: F_x_ is shown to increase when v_c_ = 1200 m/min—F_x_ ≈ 300 N (in both tested magnesium alloys) and s_tr_ = 30%—F_x_ ≈ 1000 N (in both tested magnesium alloys), compared with F_y_ for v_c_ = 1200 m/min—F_y_ ≈ 200 N (for AZ31) and F_y_ ≈ 235 N (for AZ91D), while for s_tr_ = 30%—F_y_ ≈ 313 N (for AZ31) and F_y_ ≈ 620 N (for AZ91D).
2. More stable and, therefore, effective machining, as well as lower values of the cutting forces and vibrations were obtained for the range of cutting speeds v_c_ = 1200 m/min.
3. The character of vibrations is relative to milling parameters; stable machining conditions may be obtained by increasing the cutting speed and reducing trochoidal step.
4. Dry milling of magnesium alloys can be performed in a wide range of technological parameters of machining. With the increase in v_c_, the values of the analysed machinability indicators decrease, which is an indirect confirmation that dry milling of magnesium alloys is a safe process.
5. Comparing both grades of AZ31/AZ91D magnesium alloys, it was found that
  - in general, higher values of the cutting force components prevail in AZ31 alloy milling,
  - vibration displacement x analysis shows that milling performed at lower parameters (v_c_ and s_tr_), i.e., up to 800 m/min and 20% trochoidal step, results in higher values of x in AZ31 alloy, whereas at higher cutting parameters ranges v_c_ > 800 m/min and s_tr_ > 20%—higher displacement values were recorded in AZ91D; this is potentially due to the differences in the character of the decohesion mechanism typical of the analysed workpiece material (brittle elastic or elastic–plastic) at higher v_c_ and s_tr_,
  - from analysis of vibration acceleration a, it can be seen that vibration acceleration is higher in milling of AZ91D
  - and there are no clear differences in the 3D roughness of the magnesium alloys surface AZ91D and AZ31.
6. In both tested magnesium alloys, the limit cutting speed v_c_ = 800 m/min; this limit marks the point exceeding which results in the decrease in the values of the analysed machinability parameters (the cutting force components and vibration).
7. With regard to the simulation results of the cutting forces and their correlation with actual cutting data, the analysis confirms that no discrepancies between the modelled and experimental data exceeded 15%, which is within the permissible level of error.
8. The simulation by means of artificial neural networks shows sufficient capacity and accuracy to initially determine such machining parameters as the components of the cutting force and the displacement of vibrations.
9. The relationships between nonlinear dependences between machining parameters and the values of the cutting force components or vibrations displacement x, represented in the neural structure of networks, enable investigating the process without the need for laborious, time-consuming and often cost-intensive machining tests.
10. By means of simulation, it is possible to model processes with a nonlinear course, under conditions of incomplete information about the process itself. The simulation results may be utilised in designing a tool for modelling phenomena occurring during machining, which will aid the technologist in the decision process providing them with machining parameters that ensure maintaining the stability of the process.
11. ANNs “transfer” discrete data (input data derived from tests) to continuous data (simulation data builds response surface diagrams).
12. A 50% drop in the value of 3D roughness parameters is observed at v_c_ = 1200 m/min, compared to the change as a result of modification of trochoidal step s_tr_ = 30%. The results from the tests prove that it is the cutting speed that should be increased in order to boost the efficiency and effectiveness of milling magnesium alloys.

## Figures and Tables

**Table 1:** The chemical composition (wt%) of the tested workpiece magnesium alloys.

Chemical Composition	Al	Zn	Mn	Si	Cu	Fe	Ni	Be	Mg
AZ91D	8.91	0.66	0.22	0.016	0.002	0.002	0.001	0.001	rest/other
AZ31	2.9	0.81	0.25	0.01	-	0.003	0.0004	-	rest/other

**Table 2:** Basic specifications of Kistler 9257B and amplifier 5017B [55]

9257B/5017B*	Measuring Range [kN]	Sensitivity [pC/N]	Operating Temp. Range [°C]	Measuring Range* [pC]	Linearity* [%]	Sampling Rate* [kHz]
Value	±5	≈–7.5	0–70	±10–999 000	≤±0.05	10

**Table 3:** Selected isometric images of AZ31 and AZ91D specimen surfaces after trochoidal milling.

v_c_/s_tr_	Selected Isometric Images of 3D Surface Roughness	
	AZ31	AZ91D
v_c_ 400 m/min	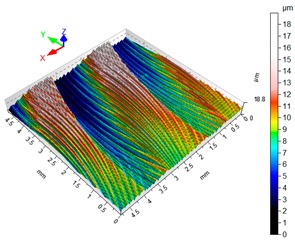	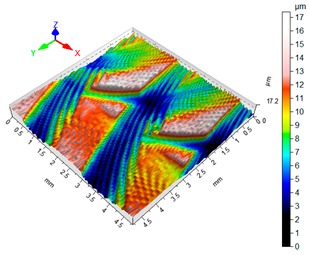
	Sa = 2.43 µm, Sz = 19.0 µm	Sa = 2.76 µm, Sz = 17.4 µm
v_c_ 1200 m/min	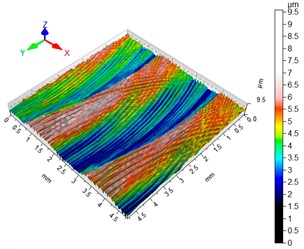	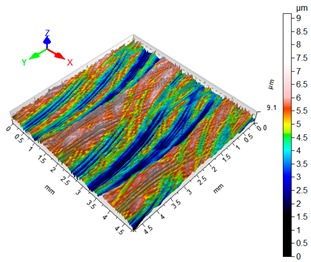
	Sa = 1.14µm, Sz = 9.58µm	Sa = 0.98 µm, Sz = 9.18 µm
s_tr_ 5%	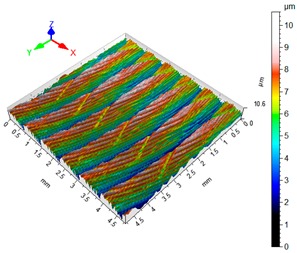	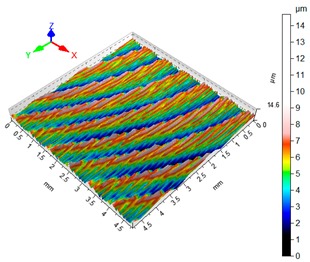
	Sa = 1.82 µm, Sz = 10.6 µm	Sa = 1.58 µm, Sz = 14.7 µm
s_tr_ 30%	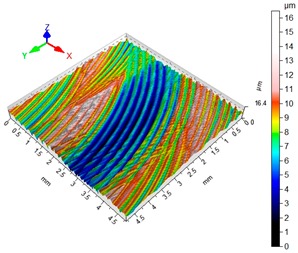	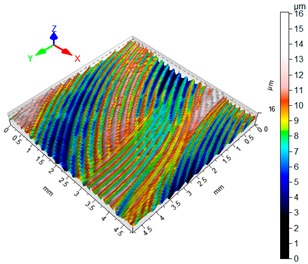
	Sa = 2.17µm, Sz = 16.5µm	Sa = 1.89 µm, Sz = 16.1µm

**Table 4:** Characteristics of multilayered perceptron (MLP) and radial basis function (RBF) networks for the cutting force components Fx, Fy and vibrations displacement x for AZ31 alloy.

Network No.	Network Name	Quality (Training, %)	Quality (Validation, %)	Error (Training)	Error (Validation)	Activation (Hidden)	Activation (Output)
Cutting force component F_x_							
1	RBF 2-2-1	92.54	86.32	403.45	573.96	Gaussian	Linear
2	RBF 2-5-1	96.88	95.23	158.67	202.54	Exponential	Sinusoidal
Cutting force component F_y_							
3	MLP 2-10-1	87.34	82.74	404.32	562.43	Linear	Tanh
4	RBF 2-6-1	99.89	89.45	25.16	43.54	Gaussian	Linear
Vibrations displacement x							
5	MLP 2-8-1	99.96	97.13	0.0001	0.0002	Tanh	Sinus
6	RBF 2-7-1	99.99	84.06	0.0001	0.0001	Gaussian	Linear

**Table 5:** Characteristics of MLP and RBF networks for the cutting force components F_x_, F_y_ and vibrations displacement x for AZ91D alloy.

Network No.	Network Name	Quality (Training, %)	Quality (Validation, %)	Error (Training)	Error (Validation)	Activation (Hidden)	Activation (Output)
Cutting force component F_x_							
1	MLP 2-7-1	95.79	89.43	387.98	422.63	Tanh	Linear
2	RBF 2-5-1	98.66	95.34	234.67	364.37	Gaussian	Linear
Cutting force component F_y_							
3	MPL 2-3-1	95.93	94.32	198.65	224.56	Exponential	Logistics
4	RBF 2-7-1	91.94	90.24	459.32	502.56	Gaussian	Linear
Vibrations displacement x							
5	MLP 2-6-1	97.88	98.84	0.0005	0.0004	Tanh	Tanh
6	RBF 2-5-1	95.97	99.43	0.0008	0.0005	Gaussian	Linear
